# Long‐Read Sequencing Reveals Ancestral intragenic *APOE* Haplotypes with Distinct Roles in Alzheimer’s Disease

**DOI:** 10.1002/alz70861_108005

**Published:** 2025-12-23

**Authors:** Agustin Ruiz, Pablo García‐González, Raquel Puerta, Amanda Cano, Clàudia Olivé, Marta Marquié, Sergi Valero, Maitee Rosende‐Roca, Pilar Sanz, Montserrat Alegret, Frederic Brosseron, Pamela Martino‐Adami, Itziar de Rojas, Michael T. Heneka, Alfredo Ramirez, Arcadi Navarro, María Eugenia Sáez, Lluís Tárraga, José E. Cavazos, Mercè Boada, Victoria Fernández, Alfredo Cabrera Socorro

**Affiliations:** ^1^ Ace Alzheimer Center Barcelona – International University of Catalunya (UIC), Barcelona Spain; ^2^ Glenn Biggs Institute for Alzheimer’s & Neurodegenerative Diseases, University of Texas Health Science Center, San Antonio, TX USA; ^3^ CIBERNED, Network Center for Biomedical Research in Neurodegenerative Diseases, National Institute of Health Carlos III, Madrid Spain; ^4^ Department of Microbiology, Immunology and Molecular Genetics, University of Texas Health Science Center, San Antonio, TX USA; ^5^ Ace Alzheimer Center Barcelona, Barcelona Spain; ^6^ German Center for Neurodegenerative Diseases (DZNE), Venusberg‐Campus 1, 53127, Bonn Germany; ^7^ Division of Neurogenetics and Molecular Psychiatry, Department of Psychiatry and Psychotherapy, Faculty of Medicine and University Hospital Cologne, University of Cologne, Cologne Germany; ^8^ Luxembourg Centre for Systems Biomedicine (LCSB), University of Luxembourg, Luxembourg Luxembourg; ^9^ BarcelonaBeta Brain Research Center (BBRC), Barcelona Spain; ^10^ CAEBI. Centro Andaluz de Estudios Bioinformáticos, Sevilla Spain; ^11^ Glenn Biggs Institute, San Antonio, TX USA; ^12^ Neurology and Physiology UT Health San Antonio, San Antonio, TX USA; ^13^ Janssen Research & Development, A Division of Janssen Pharmaceutica, Beerse, Belgium, Beerse Belgium; ^14^ Johnson & Johnson Innovative Medicine, Beerse Belgium

## Abstract

**Background:**

The apolipoprotein E (APOE) ε4 allele remains the strongest genetic risk factor for late‐onset Alzheimer’s disease (AD), yet the marked variability in its pathogenicity suggests underlying genetic complexity. Historically, efforts to resolve the intragenic architecture of APOE have been hampered by the limitations of conventional genotyping and short‐read sequencing, as well as the presence of homoplasy in common intragenic markers—misleading similarities arising from convergent variants.

**Objective:**

We leveraged Oxford Nanopore Technology (ONT) to phase intragenic APOE variants, resolve homoplasy, and examine the impact of phased haplotypes on cerebrospinal fluid (CSF) APOE protein levels and AD progression.

**Methods:**

Using long‐read sequencing in a Spanish memory clinic cohort (n = 1,267), we reconstructed full‐length 4 kb APOE haplotypes, identifying 59 unique configurations grouped into five major haplogroups. Common intragenic variants defined ancestral ε4 (4A, 4B) and ε3 (3A, 3B) haplogroups. These were analyzed for associations with CSF APOE levels (Olink platform) and progression from mild cognitive impairment (MCI) to dementia using adjusted Cox regression models.

**Results:**

ONT sequencing successfully resolved homoplasy between the APOE promoter region—particularly at rs405509—and the canonical protein isoforms, uncovering common but functionally distinct ε3A/B and ε4A/B intragenic sub‐haplotypes with independent biological effects. Carriers of the ε4A haplotype exhibited significantly lower CSF APOE protein levels (p = 0.004), whereas the ε3B haplotype was associated with elevated CSF APOE protein levels (p = 0.025). Notably, both haplotypes were linked to a slower progression from MCI to AD, independent of APOE genotype, age, sex and core CSF biomarkers.

**Conclusion:**

This study redefines the human APOE ε3 and ε4 alleles as genetically heterogeneous entities. Using ONT long‐read sequencing, we achieved high‐resolution mapping of intragenic haplotypic structure and regulatory variation previously obscured by conventional approaches. This enabled the identification of ancestral haplotypes with distinct functional profiles and potential relevance to Alzheimer’s disease pathogenesis. These findings highlight the importance of incorporating haplotype‐level resolution into Alzheimer’s risk assessment, therapeutic targeting, and precision medicine strategies.

